# A delayed-onset intracranial chronic subdural hematoma following a lumbar spinal subdural hematoma: A case report

**DOI:** 10.1097/MD.0000000000012479

**Published:** 2018-09-21

**Authors:** Takaaki Uto, Noritaka Yonezawa, Nobuhiko Komine, Yuji Tokuumi, Keiichiro Torigoe, Yukihiko Koda, Hiroyuki Tsuchiya

**Affiliations:** a Department of Orthopaedics; b Department of Neurosurgery, Asanogawa General Hospital, Ishikawa; c Department of Orthopaedic Surgery, Graduate School of Medical Sciences, Kanazawa University, Ishikawa, Japan.

**Keywords:** antiplatelet therapy, computed tomography, intracranial chronic subdural hematoma, lumbar spinal subdural hematoma, magnetic resonance imaging

## Abstract

**Rationale:**

A spinal subdural hematoma (SDH) is rarely complicated with an intracranial SDH. We found only 7 cases of spontaneous concurrent lumbar spinal and cranial SDHs, in which lumbar symptoms occurred before head symptoms.

**Patient concerns:**

We describe a 77-year-old man with spontaneous concurrent spinal and cranial SDHs, in whom the spinal SDH was identified 30 days before the intracranial chronic SDH.

**Diagnosis:**

Magnetic resonance imaging showed a spinal SDH at L4/L5. There was no paralysis, and the patient was managed conservatively. About 30 days after the onset of back pain, he experienced tinnitus and visual hallucination. Brain computed tomography showed a chronic SDH and midline shift.

**Interventions:**

Burr-hole evacuation was performed, and the patient's condition improved.

**Outcomes:**

At 5 months of follow-up, there was no recurrence of the spinal or intracranial SDH.

**Lessons:**

It is important to consider the possibility of intracranial hemorrhage when a spinal SDH is identified.

## 1 Introduction

A spinal subdural hematoma (SDH) is rare, with a reported incidence of 6.5% among all spinal hematomas.^[1,2]^ The factors associated with the development of a spinal SDH have been reported to be trauma, lumbar puncture, anticoagulation, cranial surgery, and vascular malformations.^[3]^ A spinal SDH occurring along with a cranial hematoma is extremely rare. The mechanism for the simultaneous development of spinal and cranial SDHs is unclear. To our knowledge, there are few reports on the simultaneous occurrence of intracranial and spinal SDHs, and the exact etiology is unclear. Here, we describe a case of spontaneous concurrent spinal and cranial SDHs, in which the spinal SDH was detected 30 days before the cranial SDH.

## 2 Case

**Figure 1:**
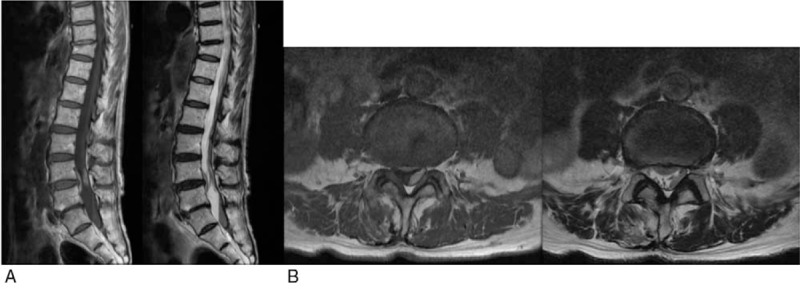
Magnetic resonance images. (A) Sagittal T1-weighted and T2-weighted images of the thoracolumbar region. (B) Axial T1-weighted and T2-weighted images at the L4/5 level.

**Figure 2:**
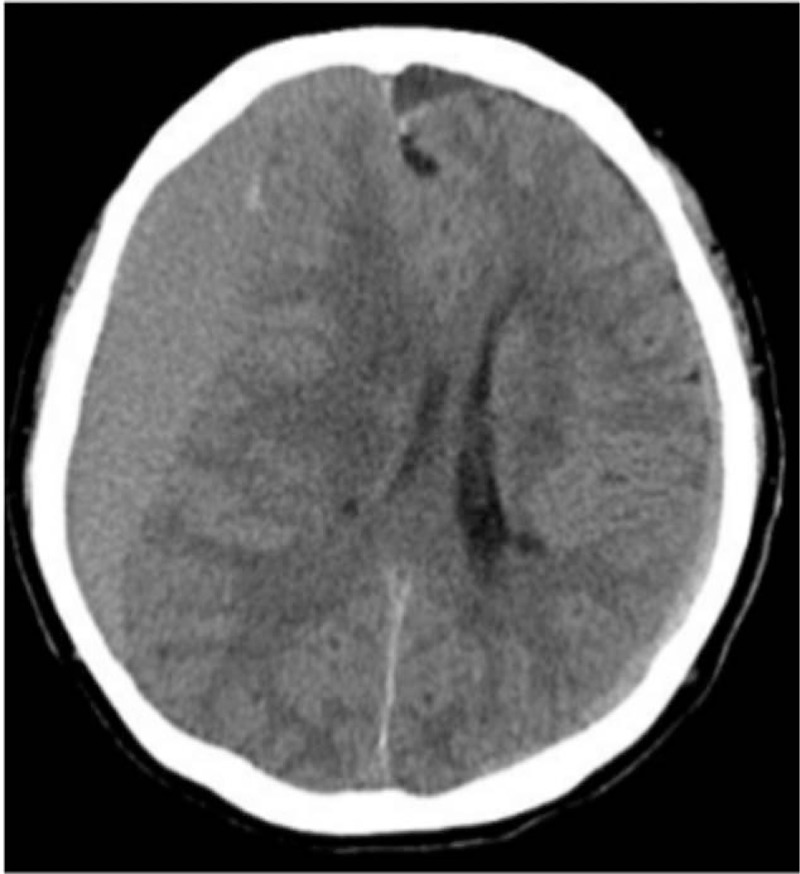
Noncontrast computed tomography after tinnitus and optical illusions shows a hematoma.

**Figure 3:**
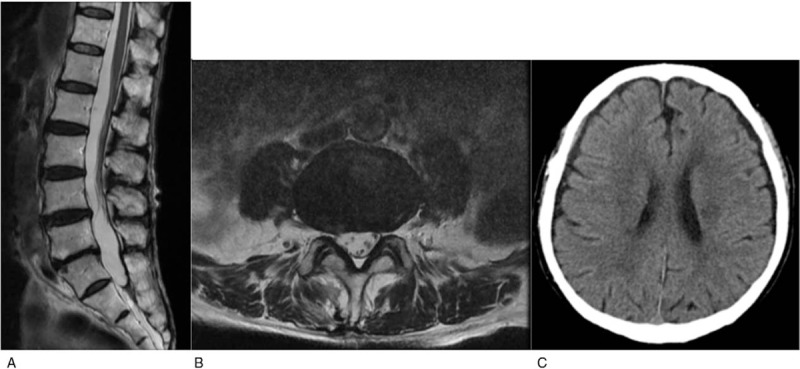
(A, B) Sagittal and axial T2-weighted spine images show complete resorption of the hematoma. (C) Non-contrast computed tomography shows significant resorption of the hematoma.

A 77-year-old man presented with an 11-day history of back pain, bilateral lower extremity pain, and numbness at the back of the right foot. He was transferred to our hospital by ambulance because of exacerbation. He had received anticoagulant and antiplatelet therapy after extrasystole and brain infarction for 2 years. There was no history of trauma or any invasive procedure. Walking gait was stable with the use of a stick. Neurological examination showed no abnormality except for a positive result in the straight-leg-raising test for the right leg. Laboratory data did not indicate coagulopathy-related diseases. We used a previously published protocol for differential diagnosis (Table 1).^[4]^ Magnetic resonance imaging (MRI) was performed 7 days after the first medical examination, and it showed a spinal SDH extending from L4 to S1 (Fig. 1). The hematomas showed high signal intensity on T1-weighted imaging and low signal intensity on T2-weighted imaging 18 days after symptom onset. The patient was treated conservatively as his neurological symptoms were not severe. We stopped anticoagulant and antiplatelet therapy, and his pain and numbness gradually improved However, 30 days after the onset of lumbar symptoms, he began experiencing tinnitus and optical illusions. Brain computed tomography (CT) showed a chronic cranial SDH and midline shift (Fig. 2). Burr-hole evacuation was performed, and his condition improved. Forty days after the surgery, brain CT showed SDH recurrence at the same location. He thus underwent another burr-hole evacuation. At 5 months of follow-up, there was no recurrence of the spinal or intracranial SDH (Fig. 3). The patient has provided informed consent for publication of the case.

**Table 1:**
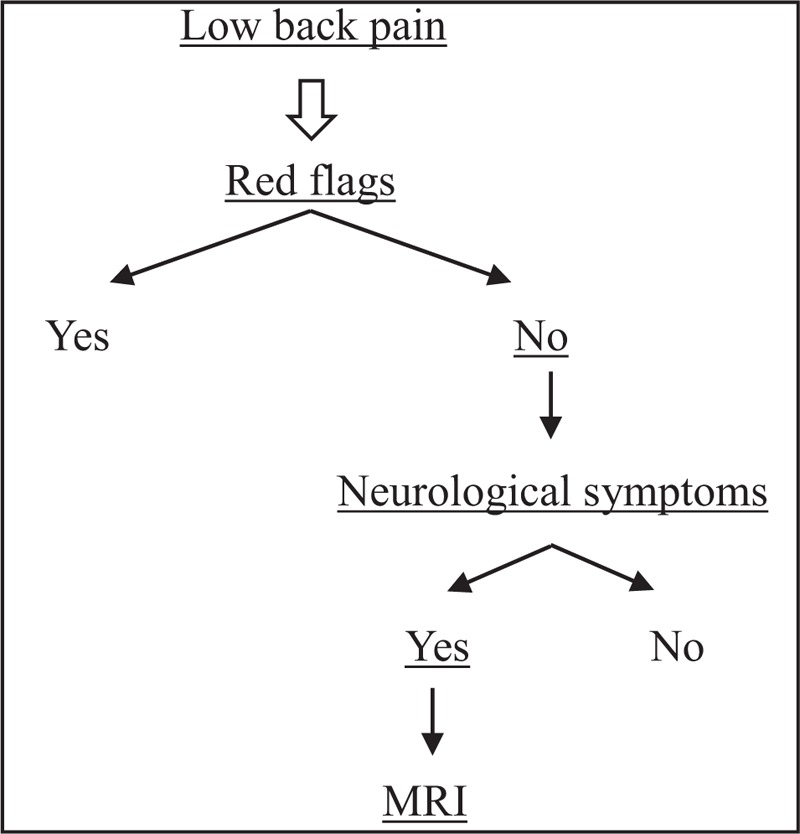
Protocol used for differential diagnosis^[4]^.

## 3 Discussion

A spinal SDH can be caused by several factors. The most common factors are blood dyscrasia, anticoagulant therapy, trauma, lumbar puncture, and vascular malformation.^[5]^ In our patient, no evidence of vascular abnormality was found on MRI. He had a history of antiplatelet and anticoagulant therapy, but he had a normal platelet count and no coagulation anomaly. Furthermore, he had no trauma or lumbar puncture. MRI is considered the best tool for detecting the presence and extent of a spinal SDH.

The treatment options of a spinal SDH depend on the clinical presentation.^[6]^ Patients with progressive neurological symptoms should be treated with surgical decompression. Our patient was initially treated conservatively because his neurological symptoms were not severe. However, he was later found to have a chronic cranial SDH.

To our knowledge, there are few reports on concurrent spinal and cranial SDHs. Kokubo et al^[7]^ reported that the rate of simultaneous occurrence of lumbar and intracranial chronic SDHs was 1.2%. However, the etiology of concurrent cranial and spinal SDHs has not been elucidated. Some reports have hypothesized that spinal SDHs might be related to progressive migration of subdural blood to the most dependent areas of the lumbosacral region.^[8]^ This theory is supported by electron microscopic observation of an anatomical continuity between the intracranial and spinal subdural spaces.^[9]^ However, other reports have suggested that a chronic cranial SDH has outer and inner membranes, and the movement of such a SDH out of the membranes has not been elucidated.^[10]^ Rebreeding or increased intracranial pressure might rupture the membranes, leading to redistribution of the hematoma to the spine.^[2]^ We assumed that primary bleeding reached the lumbar spinal region; however, after a certain point of time, bleeding remained in the cranial region.

We found only 7 cases of spontaneous concurrent lumbar spinal and cranial SDHs, in which lumbar symptoms occurred before head symptoms.^[1,2,10–14]^ In 3 of these cases, cranial MRI or CT was performed when the patients complained of lumbar symptoms, such as back pain and lower extremity numbness.^[10,11,14]^ The cranial MRI or CT revealed a cranial SDH without head symptoms. The other 4 patients complained of lumbar symptoms before head symptoms (Table 2).^[1,2,12,13]^ In previous reports, the duration between lumbar and head symptoms was within 10 days. However, in the present patient, the duration was about 30 days.

**Table 2:**
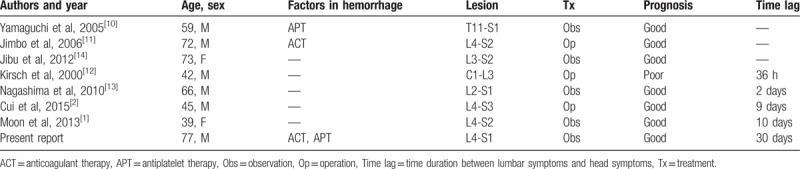
Summary of reported cases in which lumbar symptoms occurred before head symptoms.

In conclusion, we presented a rare case of spontaneous concurrent spinal and cranial SDHs. The possibility of intracranial hemorrhage should be considered when a spinal SDH is identified.

## Author contributions

**Writing – original draft:** Takaaki Uto.

**Writing – review & editing:** Yuji Tokuumi, Noritaka Yonezawa, Nobuhiko Komine, Keiichiro Torigoe, Yukihiko Koda, Hiroyuki Tsuchiya.
